# From Glycocalyx Shedding to Microvascular Collapse in Sepsis: Endothelial Pathophysiology, Organ Dysfunction, and Mechanistic Biomarkers

**DOI:** 10.3390/pathophysiology33020036

**Published:** 2026-05-29

**Authors:** Jhan S. Saavedra-Torres, Lady Viviana Acosta Castillo, Alexandra Montoya Rendon, Daniel Esteban Castro Valencia, Diego A. Lucero Guanga, Manuela Garzon Ovalle, Fabián Darío Arias Rodríguez, Andrés López-Cortés, Juan S. Izquierdo-Condoy

**Affiliations:** 1 Grupo de Investigación en Educación y Salud (GINEYSA), Facultad de Salud, Universidad Santiago de Cali, Cali 760035, Colombia; 2 Interinstitutional Group on Internal Medicine (GIMI 1), Department of Internal Medicine, Universidad Libre, Cali 760042, Colombia; 3 Facultad de Ciencias de la Salud, Corporacion Universitaria Alexander von Humboldt, Armenia 630003, Colombia; 4 Facultad de Salud, Universidad Libre, Cali 760042, Colombia; 5 Hospital Dr. Ricardo Gutierrez de La Plata, La Plata 1900, Argentina; 6 Cancer Research Group, Universidad de las Américas, Quito 170124, Ecuador; 7 One Health Research Group, Universidad de Las Américas, Quito 170124, Ecuador

**Keywords:** sepsis, endothelial dysfunction, glycocalyx, syndecan-1, microvascular coagulopathy

## Abstract

Sepsis is a systemic disorder in which infection-induced inflammation progressively disrupts vascular homeostasis and drives organ dysfunction. This review reframes septic pathophysiology as a sequential and self-amplifying process centered on endothelial failure. Early activation of innate immune pathways by pathogen- and damage-associated molecular patterns promotes cytokine release, oxidative stress, and enzymatic degradation of the endothelial glycocalyx. Loss of this protective surface layer exposes endothelial cells to unbuffered inflammatory and mechanical injury, impairing mechanotransduction, increasing leukocyte and platelet adhesion, and destabilizing vascular barrier function. Subsequent disruption of intercellular junctions promotes capillary leakage, tissue edema, and impaired oxygen diffusion, while mitochondrial dysfunction and redox imbalance reduce endothelial repair capacity. In parallel, complement activation, neutrophil extracellular trap formation, platelet–leukocyte interactions, and loss of anticoagulant signaling shift the microvasculature toward a prothrombotic and proinflammatory state. These interconnected mechanisms culminate in microvascular incoherence, characterized by heterogeneous capillary flow, regional hypoxia, impaired oxygen extraction, and progressive organ failure despite apparent restoration of systemic hemodynamics. Within this framework, biomarkers such as syndecan-1, soluble thrombomodulin, angiopoietin-2, von Willebrand factor, and plasminogen activator inhibitor-1 are best interpreted as mechanistic readouts of glycocalyx shedding, endothelial injury, permeability imbalance, and thromboinflammatory activation. Understanding sepsis as an evolving endothelial pathophysiological process provides a coherent framework for integrating inflammation, vascular leakage, hypoxia, coagulation, and organ dysfunction while identifying mechanistic biomarkers that reflect distinct stages of microvascular collapse.

## 1. Introduction

Sepsis is increasingly understood as a state of systemic immunodisruption, defined not only by excessive inflammation but also by a breakdown in coordination among three interdependent axes: inflammatory signaling, redox balance, and cellular metabolism [1,2,3,4,5,6,7]. At the molecular level, pathogen- and damage-associated molecular patterns activate pattern-recognition receptors, including Toll-like receptor 4, initiating nuclear factor kappa B-dependent cytokine programs involving interleukin-6 and tumor necrosis factor [2,8,9]. In parallel, mitochondrial overproduction of reactive oxygen species, together with depletion of antioxidant reserves such as glutathione, disrupts redox homeostasis and promotes oxidative injury to lipids, proteins, and DNA [10,11]. This process is accompanied by a metabolic shift from oxidative phosphorylation toward aerobic glycolysis, a Warburg-like phenotype that reduces adenosine triphosphate efficiency and compromises cellular resilience [1,2,7,12,13].

Despite substantial advances in critical care, sepsis continues to affect more than 30 million individuals each year, with mortality rates ranging from 20% to more than 50% in severe cases [14,15]. These outcomes underscore that disease progression is driven not only by pathogen burden but also by host-response heterogeneity [5,6,7]. Biologically, this heterogeneity reflects differences in immune activation, endothelial integrity, metabolic adaptability, and the capacity to restore tissue homeostasis [14,15]. Clinically, it supports the need for earlier risk stratification, continuous monitoring, and individualized interventions that integrate timely antimicrobial therapy with host-response assessment [15,16].

Sepsis also extends beyond the acute phase. A substantial proportion of survivors develop long-term sequelae consistent with post-sepsis syndrome, reinforcing its nature as a multisystem disorder with persistent biological and functional consequences [17,18]. Sustained immune dysregulation, endothelial injury, and metabolic reprogramming may contribute to long-term organ dysfunction, impaired recovery, and reduced quality of life [17,18].

This variability has challenged inflammation-centered paradigms and shifted attention toward the microcirculation as a determinant of disease progression. Under physiological conditions, the vascular endothelium functions as a dynamic regulatory interface that preserves tissue perfusion, controls leukocyte trafficking, maintains barrier integrity, and balances permeability, anticoagulant activity, and microcirculatory stability through nitric oxide-dependent signaling and an intact glycocalyx [19,20,21,22,23,24]. During sepsis, this equilibrium is rapidly disrupted when pathogen- and damage-associated molecular patterns engage endothelial receptors, activating nuclear factor kappa B and mitogen-activated protein kinase pathways. These cascades promote cytokine release, oxidative stress, glycocalyx degradation, and intercellular junction destabilization [1,6,25,26].

Within this framework, endothelial dysfunction should be interpreted as an active driver of septic pathophysiology rather than a passive consequence of systemic inflammation [27,28]. Loss of endothelial homeostasis promotes vascular leakage, coagulation imbalance, impaired oxygen diffusion, and microvascular instability, creating a self-sustaining cycle of tissue hypoxia and organ dysfunction [15,17,19,20,23,24]. Biomarkers such as syndecan-1 and soluble thrombomodulin reflect this transition by linking glycocalyx shedding and endothelial injury to disease severity and unfavorable clinical trajectories [21,22,23,24].

Despite its central role, endothelial dysfunction remains underrecognized as a unifying mechanism in sepsis. Current approaches often prioritize inflammation control and systemic hemodynamic stabilization while overlooking the vascular compartment where critical injury unfolds [15,17]. This review reframes sepsis as an immunovascular failure driven by glycocalyx loss, junctional disruption, oxidative injury, thromboinflammation, and microcirculatory collapse. By positioning the endothelium as a central integrator of systemic injury, this perspective provides a pathophysiological framework for understanding organ failure, interpreting mechanistic biomarkers, and identifying therapeutic strategies aimed at preserving vascular coherence.

## 2. Methods

To enhance transparency and reproducibility, this review was conducted using a structured narrative approach informed by systematic search principles. A comprehensive literature search was performed in PubMed/MEDLINE, Scopus, and Web of Science for studies published between January 2000 and December 2025.

The search strategy combined controlled vocabulary and free-text terms related to sepsis, endothelial dysfunction, glycocalyx degradation, microcirculation, vascular permeability, thromboinflammation, immunothrombosis, oxidative stress, mitochondrial dysfunction, and endothelial biomarkers. Additional targeted searches were conducted to identify emerging literature on systems biology, immunovascular integration, organ-on-chip models, endothelial repair, and endothelium-targeted therapeutic strategies.

Eligible sources included original research articles, translational studies, experimental models, observational clinical studies, and high-quality reviews that provided mechanistic insight into endothelial biology in sepsis or related forms of critical illness. Studies were prioritized when they contributed to understanding interactions among inflammatory, coagulative, metabolic, redox, and microvascular pathways. Purely descriptive reports without mechanistic relevance were excluded. Reference lists of key articles were manually screened to identify additional relevant publications.

This review was not designed as a systematic review or meta-analysis. Instead, the objective was to construct a mechanism-driven synthesis integrating preclinical, translational, and clinical evidence into a unified pathophysiological framework. This approach allowed the convergence of heterogeneous evidence domains to be interpreted in relation to endothelial dysfunction, microvascular collapse, mechanistic biomarkers, and therapeutic implications in sepsis.

## 3. The Endothelium as a Central Regulator of Septic Pathophysiology

Sepsis can be conceptualized as a hierarchically organized failure of the immunovascular system in which the endothelium functions as a central regulatory interface rather than a passive target of systemic inflammation. Multiple pathways that may appear independent, including NETosis, complement activation, angiopoietin-2/Tie2 imbalance, sphingosine-1-phosphate depletion, von Willebrand factor release, and pathogen-derived mediators such as NS1, ultimately converge on endothelial integrity as a major determinant of disease progression [23,29,30,31,32,33]. Early glycocalyx degradation exposes the endothelial surface, increases leukocyte and platelet adhesion, and initiates a shift toward a proinflammatory and procoagulant phenotype [18,23].

These convergent mechanisms amplify endothelial dysfunction through mutually reinforcing pathways. Excessive NETosis promotes direct cytotoxicity and microvascular obstruction, whereas insufficient NET formation may compromise microbial containment and favor persistent infection [34,35]. Complement activation further intensifies vascular injury, particularly through excessive C5a signaling, which promotes inflammation, endothelial damage, and immune exhaustion [2,18]. In parallel, angiopoietin-2 antagonizes Tie2-mediated vascular stabilization, increasing junctional instability and permeability [22,36]. Depletion of sphingosine-1-phosphate weakens barrier integrity, while von Willebrand factor release promotes platelet aggregation and capillary obstruction [2,6,21,28]. Together, these alterations transform the endothelium into an active amplifier of inflammation, coagulation, and microvascular instability [4,37,38].

Within this framework, sepsis severity is not dictated solely by pathogen burden or inflammatory magnitude, but by the capacity of the endothelium to preserve functional coherence under sustained stress [39,40]. Under physiological conditions, endothelial cells coordinate permeability, perfusion, hemostasis, leukocyte trafficking, and vascular tone. Once this coordination fails, the system shifts from adaptive host defense to self-sustaining vascular instability, clinically expressed as vasoplegia, capillary leak, prothrombotic activation, and progressive organ dysfunction [41,42,43]. Systems biology analyses support this interpretation by showing convergence across inflammatory, coagulative, and metabolic axes, generating emergent dysfunction that cannot be explained by isolated pathways alone [44,45].

Endothelial failure evolves through interdependent modules. Glycocalyx degradation represents the initial structural injury, removing a protective layer that buffers mechanical forces and regulates cellular interactions [4,46,47]. Junctional disruption then converts this structural vulnerability into functional hyperpermeability, allowing uncontrolled transvascular movement of fluids and macromolecules [48,49]. Dysregulation of homeostatic signaling axes, including angiogenic and lipid pathways, further prevents restoration of endothelial stability and has been linked to progression toward shock and sustained organ impairment [50,51]. Finally, complement activation, platelet–leukocyte interactions, and NET formation reinforce a proinflammatory and procoagulant endothelial phenotype, contributing to diffuse microthrombosis and persistent microvascular dysfunction [37,49,51].

The clinical consequence is the progressive loss of microcirculatory coherence. Glycocalyx loss impairs mechanotransduction and endothelial responsiveness to flow, reducing nitric oxide bioavailability and altering vasoregulation [52,53]. Junctional disruption promotes capillary leakage, interstitial edema, and increased diffusion distance for oxygen [54,55]. Amplifying thromboinflammatory circuits transform the endothelium into a proadhesive surface that promotes cellular aggregation, localized coagulation, and signal propagation [15,19]. These processes are synergistic rather than additive, forming feedback loops in which barrier failure intensifies inflammation, and inflammation accelerates vascular breakdown.

As endothelial dysfunction progresses, the microcirculation loses its ability to distribute flow homogeneously. Regions of preserved perfusion coexist with areas of stagnation and shunting, producing heterogeneous oxygen delivery despite apparently corrected systemic hemodynamics [56,57,58,59]. This explains a central paradox of sepsis: normalization of blood pressure or cardiac output does not necessarily restore tissue oxygenation. At this stage, cellular injury becomes increasingly uncoupled from the initial pathogen and instead reflects the failure of the host vascular network to recover internal regulation [60,61].

This persistent instability helps explain why organ dysfunction may progress despite adequate antimicrobial therapy. In advanced sepsis, inflammation, coagulation, redox imbalance, and metabolic dysfunction become tightly coupled, generating a network state that is difficult to reverse [17,54]. Experimental and translational evidence further indicates that endothelial injury may persist beyond the acute phase, with impaired vasodilation, enhanced vasoconstriction, reduced nitric oxide bioavailability, platelet-derived extracellular vesicle amplification, NET formation, and endothelial senescence programs contributing to sustained vascular dysfunction [18,62,63,64,65,66].

Accordingly, the endothelial cell defines a critical threshold between adaptive host response and functional collapse [15,16]. Sepsis should therefore not be viewed as a simple sum of inflammatory, infectious, and coagulative events, but as an emergent state in which the immunovascular network loses its capacity for self-regulation [67,68]. This perspective provides a coherent mechanistic foundation for interpreting endothelial biomarkers, understanding persistent organ dysfunction, and developing therapeutic strategies aimed at preserving or restoring microvascular integrity [66,69].

### 3.1. Disseminated Intravascular Coagulation as an Immunothrombotic Endpoint of Endothelial Collapse

Within the septic immunovascular environment, disseminated intravascular coagulation represents the transition from regulated hemostatic adaptation to diffuse microvascular disorganization driven by endothelial collapse [70,71]. Rather than functioning as a secondary coagulation abnormality, sepsis-associated DIC reflects a systems-level failure in which inflammatory signaling, endothelial activation, platelet recruitment, and coagulation become pathologically synchronized within the microcirculation [6,70,71]. Sustained expression of tissue factor, uncontrolled thrombin amplification, and progressive depletion of endogenous anticoagulant mechanisms establish a vascular state characterized by intraluminal fibrin accumulation, endothelial surface remodeling, and loss of capillary flow adaptability [72,73]. Simultaneously, suppression of fibrinolytic activity through increased plasminogen activator inhibitor-1 expression promotes persistence of fibrin-rich microdomains that obstruct tissue perfusion and intensify regional hypoxia. This process extends beyond thrombosis itself and profoundly alters endothelial bioenergetic stability, oxygen diffusion dynamics, and vasoregulatory responsiveness, particularly within metabolically vulnerable organs such as the lungs, kidneys, liver, and myocardium [72,74]. At the cellular level, endothelial phenotypic reprogramming is accompanied by Weibel–Palade body exocytosis, excessive von Willebrand factor release, platelet tethering, and progressive disruption of vascular autoregulatory integrity. The resulting vascular landscape is not merely procoagulant, but structurally and functionally incapable of maintaining homogeneous microcirculatory exchange [74,75,76]. In this context, disseminated intravascular coagulation should be understood as a terminal expression of endothelial immunothrombotic failure, where inflammation, coagulation, hypoxia, and vascular dysfunction converge into a self-amplifying network of progressive microvascular instability [53,68].

### 3.2. Endothelial Exhaustion and Perfusion Failure

As disseminated intravascular coagulation progresses, the septic microcirculation evolves from a dynamically regulated perfusion network toward a state of fixed vascular dysfunction and impaired tissue oxygen delivery [77,78]. Sustained endothelial activation progressively disrupts the ability of capillary beds to adapt to regional metabolic requirements, generating heterogeneous perfusion patterns characterized by intermittent flow, capillary stagnation, and defective oxygen extraction [78,79]. This process is accompanied by endothelial functional exhaustion, reflected by reduced nitric oxide bioavailability [77,80], intracellular bioenergetic impairment, and diminished responsiveness to vasoregulatory signaling [80,81,82]. Concurrently, persistent platelet–endothelium interaction and fibrin-rich microvascular aggregates increase intraluminal resistance and further compromise capillary perfusion. Unlike isolated thrombotic disorders, sepsis-associated disseminated intravascular coagulation produces a profound dissociation between systemic hemodynamic restoration and effective cellular oxygen utilization, allowing for persistent tissue hypoxia and mitochondrial dysfunction despite apparently adequate macrovascular parameters [68,77]. Experimental and translational evidence further suggests that this microvascular rigidity promotes endothelial senescence, defective barrier restitution, and sustained inflammatory amplification within highly perfused organs [83,84]. Consequently, disseminated intravascular coagulation should be interpreted as a progressive failure of endothelial adaptive physiology culminating in advanced microvascular perfusion collapse [85,86].

## 4. Glycocalyx Degradation as an Initiating Event

Glycocalyx degradation represents one of the earliest structural events in septic endothelial dysfunction. Under physiological conditions, the endothelial glycocalyx forms a dynamic luminal surface layer that buffers mechanical forces, regulates mechanotransduction, limits inappropriate leukocyte and platelet adhesion, and contributes to permeability control [1,4,6,25,26,46,47]. Its disruption therefore removes a critical protective interface between circulating inflammatory mediators and the endothelial cell surface, exposing the vasculature to unbuffered biochemical and mechanical stress [1,19,20].

During sepsis, pathogen- and damage-associated inflammatory signals promote oxidative stress and induce glycocalyx-degrading enzymes, including matrix metalloproteinases, heparanases, and hyaluronidases [19,20,36,87]. These enzymatic and redox-mediated processes fragment the glycocalyx and release its components into the circulation. Circulating syndecan-1 and hyaluronic acid therefore serve as measurable indicators of endothelial surface layer injury and have been associated with increased vascular permeability, disease severity, and unfavorable outcomes in septic patient [87,88,89,90,91,92].

The functional consequences of glycocalyx shedding are immediate and multifactorial. Loss of the endothelial surface layer impairs mechanotransduction, alters shear-stress sensing, and weakens the capacity of endothelial cells to regulate vascular tone and barrier function [4,46,47]. At the same time, exposure of the endothelial membrane facilitates leukocyte and platelet adhesion, complement activation, and transition toward a proinflammatory and procoagulant phenotype [1,2,4,19,31]. Although these responses may initially contribute to host defense, their persistence promotes microvascular obstruction, capillary leak, interstitial edema, and impaired oxygen diffusion.

Glycocalyx injury also creates a self-amplifying inflammatory loop. Shed glycocalyx fragments can act as damage-associated molecular signals, reinforcing local inflammation and further accelerating endothelial activation [1,19,20]. As structural damage and immune activation progress in parallel, the endothelium loses its ability to maintain vascular homeostasis, linking early surface layer degradation to downstream junctional disruption, thromboinflammation, and microcirculatory failure.

From a therapeutic perspective, glycocalyx preservation remains biologically attractive but clinically incompletely validated. Careful fluid administration may reduce hydrostatic overload and limit atrial natriuretic peptide-associated glycocalyx shedding, although restrictive fluid strategies have produced heterogeneous clinical results [87,90,91]. Adjunctive vasopressin may reduce catecholamine exposure while supporting vascular tone, and moderate glycemic control may indirectly protect the endothelial surface by limiting reactive oxygen species generation, RAGE activation, and NET formation [87,90,91]. Colloidal therapies such as fresh frozen plasma or albumin have a plausible mechanistic rationale through provision of protease inhibitors and sphingosine-1-phosphate, but clinical outcomes remain inconsistent [46,87]. Antithrombin supplementation is also pathophysiologically relevant because it combines anticoagulant activity with potential stabilization of heparan sulfate chains within the endothelial surface layer [87,90,91]. Overall, these strategies should be interpreted as supportive or investigational approaches aimed at limiting glycocalyx degradation rather than as established endothelial-restorative therapies.

## 5. Junctional Disruption and Vascular Leakage

Junctional disruption translates septic endothelial injury into functional vascular leakage. After glycocalyx degradation exposes the endothelial surface, inflammatory mediators and oxidative stress destabilize intercellular junctions, particularly adherens junctions that maintain barrier integrity. This process increases capillary permeability, allowing uncontrolled extravasation of fluid and plasma proteins into the interstitial space [37,83,93]. The resulting edema increases the diffusion distance for oxygen, impairs tissue oxygenation, and contributes to organ dysfunction even when systemic hemodynamic variables appear corrected.

The consequences of vascular leakage vary across organs according to local endothelial architecture and metabolic demand. In the lungs, disruption of the alveolar–capillary barrier causes protein-rich alveolar flooding, impaired gas exchange, reduced lung compliance, and progression toward acute respiratory distress syndrome. In the kidneys, endothelial injury and microvascular dysfunction compromise glomerular filtration and contribute to septic acute kidney injury. In the liver, sinusoidal hyperpermeability facilitates leakage of albumin and inflammatory mediators, promoting hepatocellular stress and amplification of local inflammation. In the heart, microvascular dysfunction and altered vascular tone impair coronary perfusion and contribute to septic cardiomyopathy [37,83,93]. By contrast, tissues with tighter junctional architecture, such as the blood–brain barrier, or lower pressure gradients, such as adipose tissue, may show relatively delayed involvement [37,83,93].

Experimental models of septic pulmonary endothelial injury suggest that barrier failure follows a biphasic pattern. In the early phase, proinflammatory cytokines such as tumor necrosis factor alpha, interleukin-1 beta, and interferon gamma induce cytoskeletal retraction and junctional disassembly, producing a rapid decrease in transendothelial electrical resistance and increased macromolecular leakage into the interstitial and alveolar spaces [94,95]. This early increase in permeability appears to be largely apoptosis-independent, as markers of programmed cell death, including caspase activation, mitochondrial membrane depolarization, and DNA fragmentation, typically emerge later, between 8 and 16 h after inflammatory exposure [94,96].

The late phase is characterized by impaired barrier recovery. Although apoptosis does not appear to initiate the early permeability defect, it contributes to endothelial cell detachment, delayed junctional repair, and persistence of vascular leakage [94,96]. Experimental inhibition of caspase activation does not prevent the initial fall in transendothelial electrical resistance, but it partially restores barrier resistance and reduces permeability at later time points, supporting the concept that apoptosis mainly prolongs the edematous phase rather than triggering the initial barrier collapse [94,96].

**Figure 1:**
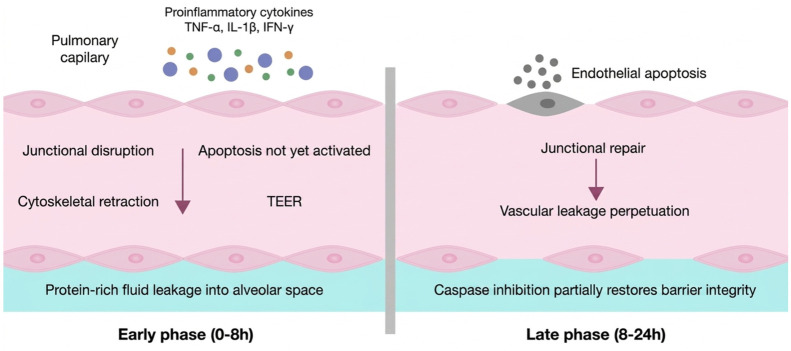
Biphasic endothelial response to sepsis-induced pulmonary injury. Sepsis induces a biphasic disruption of the pulmonary microvascular barrier. During the early phase (0–8 h), proinflammatory cytokines (TNF-α, IL-1β, IFN-γ) trigger junctional disassembly and cytoskeletal retraction, leading to decreased transendothelial electrical resistance (TEER) and protein-rich fluid leakage into the alveolar space. Apoptosis is not yet activated. In the late phase (8–24 h), endothelial apoptosis becomes prominent, impairing junctional repair and perpetuating vascular leakage. Caspase inhibition during this phase partially restores endothelial integrity and facilitates barrier recovery.

This biphasic model clarifies how septic vascular leakage evolves from an early, potentially reversible phase dominated by cytoskeletal contraction and junctional disassembly to a later phase in which endothelial apoptosis and defective repair sustain edema. Pathophysiologically, this transition links acute barrier destabilization to persistent organ dysfunction. Therapeutically, it suggests that early strategies should focus on stabilizing endothelial junctions and limiting cytoskeletal contraction, whereas later interventions may need to preserve endothelial viability and promote barrier repair [94,96] (Figure 1).

## 6. Oxidative Stress, Mitochondrial Dysfunction, and Impaired Repair

Oxidative stress and mitochondrial dysfunction are central amplifiers of septic endothelial injury. Once inflammatory and mechanical stress destabilize the endothelial barrier, excessive reactive oxygen and nitrogen species impair cellular repair, disrupt cytoskeletal organization, and perpetuate vascular leakage. In this context, TRPM7/TRPV4 activation and VEGF-related signaling should be interpreted as context-dependent modulators of endothelial behavior rather than universal primary drivers of dysfunction [97,98,99]. Their pathophysiological relevance emerges when the endothelial system is already destabilized and exposed to convergent mechanical stress, inflammatory signaling, and metabolic imbalance [68,97].

TRPV4 activation translates mechanical stimuli, including shear stress and stretch, into calcium influx, directly influencing cytoskeletal tension and junctional stability [100,101]. TRPM7 integrates ionic homeostasis with intracellular signaling, thereby affecting endothelial survival, redox balance, and barrier maintenance. In parallel, VEGF signaling regulates endothelial permeability and survival through receptor-mediated pathways that influence junctional proteins and cytoskeletal remodeling [97,101]. These pathways are closely linked to glycocalyx integrity, oxidative stress, and immunothrombotic activation, meaning that their effects depend on timing, intensity, and the broader state of endothelial injury.

Mitochondrial dysfunction further weakens endothelial resilience. In sepsis, inflammatory mediators and oxidative stress impair mitochondrial quality control, reduce bioenergetic efficiency, and limit the capacity of endothelial cells to restore barrier integrity after injury. This contributes to persistent redox imbalance, defective junctional repair, and sustained microvascular instability [102,103]. Autophagy is one of the key adaptive mechanisms through which endothelial cells attempt to remove damaged organelles, maintain mitochondrial function, and regulate inflammatory signaling. However, its effects are highly context-dependent: insufficient autophagy may permit accumulation of damaged mitochondria and reactive oxygen species, whereas excessive or poorly timed activation may interfere with host defenses and cellular recovery [102,103,104,105].

Experimental evidence supports this dual role. In a rat model of cecal ligation and puncture, sepsis induced early renal endothelial injury accompanied by activation of autophagic machinery in glomerular endothelial cells, reflected by increased LC3-II expression and the appearance of autophagosomes [104,106,107,108]. Administration of rapamycin before sepsis induction further increased the LC3-II/LC3-I ratio and autophagic vesicle formation. These changes were associated with transient reductions in soluble thrombomodulin and partial restoration of VE-cadherin expression in renal tissue, suggesting improved preservation of endothelial junctional integrity [104,106,107,108].

Histologically, rapamycin-associated autophagy was linked to reduced glomerular capillary dilation, less hypervascularization, decreased interstitial edema, and diminished leukocyte infiltration in the renal cortex [104,107,108]. Sepsis also increased expression of BMP and activin membrane-bound inhibitor, a pseudoreceptor that negatively regulates transforming growth factor beta and Wnt/β-catenin signaling. Rapamycin-induced autophagy transiently normalized this response through targeted degradation, suggesting that selective removal of maladaptive signaling intermediates may contribute to improved endothelial cohesion and resistance to inflammatory stress [104,107,108].

However, these structural improvements did not translate into significant recovery of renal function, as assessed by serum creatinine and proteinuria, nor did they improve 48-h survival after cecal ligation and puncture [104]. This finding is important because it indicates that endothelial autophagy can attenuate local vascular injury without fully reversing septic acute kidney injury or systemic disease progression. Therefore, autophagy should be interpreted as a protective but incomplete endothelial stress response rather than a stand-alone therapeutic solution.

Overall, oxidative stress, mitochondrial dysfunction, calcium-dependent signaling, VEGF-related permeability regulation, and autophagy form an interconnected repair–injury axis in septic endothelium. These mechanisms determine whether endothelial cells recover barrier function or remain locked in a state of persistent dysfunction. Therapeutically, selective enhancement of endothelial repair pathways, including autophagy modulation, remains promising but primarily experimental and requires careful attention to timing, disease stage, organ specificity, and preservation of antimicrobial host defense [104,106,107,108].

## 7. Thromboinflammation, NETosis, Complement, and Microthrombosis

Inflammation is essential for pathogen clearance, but in sepsis, its loss of proportionality and temporal control becomes a major driver of endothelial injury [15,19,109]. The central defect is therefore not inflammation itself, but the transition from a coordinated antimicrobial response to a self-amplifying inflammatory state that damages the host microvasculature. Although antibiotics reduce pathogen burden, they do not restore the host’s capacity to regulate immune activation, endothelial stability, and microvascular perfusion [64,110]. Complete suppression of inflammation is neither feasible nor desirable as it would compromise phagocytosis, oxidative killing, antigen presentation, and other essential antimicrobial functions. Conversely, uncontrolled inflammatory amplification disrupts endothelial integrity, increases permeability, activates coagulation, and accelerates organ dysfunction [103,111].

This imbalance provides the foundation for thromboinflammation, a process in which inflammatory and coagulative pathways become tightly coupled within the microvasculature. Activated endothelial cells increase adhesion molecule expression, recruit leukocytes and platelets, and shift toward a procoagulant phenotype. At the same time, inflammatory mediators amplify tissue factor expression, suppress endogenous anticoagulant pathways, and promote platelet–leukocyte interactions. The result is a microvascular environment in which immune activation and coagulation reinforce each other, generating localized thrombosis, impaired capillary flow, and progressive tissue hypoxia [112,113,114].

Neutrophil extracellular traps represent a key component of this thromboinflammatory response. When appropriately regulated, NET formation contributes to pathogen containment; however, excessive or persistent NETosis injures endothelial cells, exposes prothrombotic surfaces, and promotes microvascular obstruction [115,116]. NET-associated proteases, histones, and reactive oxygen species can degrade endothelial structures, destabilize junctions, and intensify vascular leakage. In parallel, NETs provide scaffolds for platelet adhesion and fibrin deposition, thereby linking innate immune activation to microthrombus formation and capillary flow impairment.

Complement activation further amplifies this process. In sepsis, excessive complement signaling promotes leukocyte recruitment, endothelial activation, and vascular injury while also interacting with coagulation pathways to intensify microthrombosis. This creates a feed-forward loop in which complement activation, NETosis, platelet aggregation, and endothelial dysfunction converge to obstruct the microcirculation and perpetuate organ injury [113,114].

Calcium-dependent signaling also contributes to this inflammatory–vascular interface. Transient receptor potential channels, particularly TRPV4 and TRPM7, regulate calcium influx in endothelial and immune cells and influence vascular permeability, cytokine signaling, and leukocyte behavior [117,118]. In experimental models, activation of these channels increases intracellular calcium, promotes endothelial contraction, disrupts VE-cadherin-mediated junctional stability, and enhances capillary leakage [115,118]. In immune cells, TRP channels modulate neutrophil activation, migration, and reactive oxygen species generation, linking calcium signaling to both antimicrobial defense and collateral tissue injury [115,116].

Despite their mechanistic relevance, TRP channels remain difficult therapeutic targets. Their broad expression across vascular, renal, pulmonary, and immune compartments raises concern for off-target effects, and most supporting evidence remains preclinical [7,115]. Therefore, TRP signaling is best interpreted as a context-dependent amplifier of endothelial permeability and immune activation rather than as an isolated therapeutic target.

Overall, thromboinflammation reflects the failure to maintain an intermediate inflammatory tone sufficient for pathogen elimination but restrained enough to preserve endothelial integrity. Once this balance is lost, NETosis, complement activation, platelet–leukocyte interactions, calcium-dependent signaling, and coagulation converge to produce microthrombosis, capillary obstruction, and persistent tissue hypoxia. This mechanism links immune dysregulation directly to microvascular collapse and provides a pathophysiological bridge between infection control, endothelial injury, and organ failure [112,113,114].

## 8. Microvascular Incoherence, Hypoxia, and Organ Dysfunction

Glycocalyx loss, junctional disassembly, oxidative injury, and thromboinflammatory activation collectively transform the microvasculature from a regulated exchange network into a driver of tissue hypoxia and organ dysfunction [15,18,20,21]. Once endothelial integrity is lost, capillary flow becomes spatially heterogeneous, with areas of no-flow or stagnation adjacent to regions of preserved or excessive perfusion. This phenomenon, often described as microvascular shunting, disrupts effective oxygen delivery despite apparently adequate systemic circulation [47,87,119,120].

This loss of microvascular coherence explains one of the central paradoxes of sepsis: normalization of systemic hemodynamic variables does not necessarily restore tissue oxygenation. Even when cardiac output and arterial pressure are corrected, oxygen delivery at the cellular level may remain impaired because of capillary obstruction, endothelial swelling, microthrombosis, increased diffusion distance, and altered oxygen extraction [13,34,68]. As a result, tissue hypoxia may persist despite conventional resuscitation, marking a transition from reversible vascular dysfunction toward progressive multiorgan failure [31,121,122,123].

At the molecular level, sustained endothelial activation promotes a prothrombotic phenotype. Upregulation of tissue factor, suppression of the protein C pathway, and reduced expression of tissue factor pathway inhibitor shift the hemostatic balance toward thrombin generation and fibrin deposition [34,68]. Simultaneously, release of von Willebrand factor from Weibel–Palade bodies enhances platelet adhesion and aggregation, facilitating microthrombus formation within the capillary bed [47,87,119,120]. These microthrombi further impair perfusion and act as platforms for leukocyte recruitment, thereby reinforcing local inflammation and vascular obstruction.

Cytoskeletal remodeling further contributes to this process. Dysregulation of small GTPases, including activation of RhoA and suppression of Rac1, promotes actin–myosin contraction and destabilizes endothelial junctions. Increased activity of myosin light-chain kinase and Rho kinase widens paracellular gaps, facilitating plasma leakage, leukocyte extravasation, and tissue edema [14,21,88,124]. In parallel, oxidative stress generated by NADPH oxidase activation and endothelial nitric oxide synthase uncoupling produces reactive oxygen and nitrogen species, including peroxynitrite. These mediators damage membrane lipids, cytoskeletal proteins, and mitochondrial components, impairing endothelial survival, limiting repair capacity, and sustaining vascular dysfunction.

The functional endpoint of these mechanisms is microcirculatory incoherence, characterized by heterogeneous capillary flow, regional hypoxia, impaired oxygen extraction, and progressive organ injury despite normalized systemic hemodynamics. At this stage, tissue damage becomes increasingly decoupled from pathogen burden and instead reflects collapse of vascular self-regulation [41,42,125]. The microcirculation no longer adapts effectively to metabolic demand, and oxygen delivery becomes uncoupled from tissue utilization.

Although this process affects multiple organs, the pulmonary microvasculature is particularly vulnerable. The alveolar–capillary interface becomes exposed to intense inflammatory and thrombotic activity, resulting in protein-rich edema, surfactant dilution, reduced lung compliance, and impaired gas exchange. Microvascular thrombosis further worsens ventilation–perfusion mismatch, creating areas of functional shunt and refractory hypoxemia [47,87,119,120]. Clinically, this process contributes to acute respiratory distress syndrome, a major manifestation of sepsis-related microvascular failure and a substantial contributor to mortality in critical illness [31,121,122,123].

**Figure 2:**
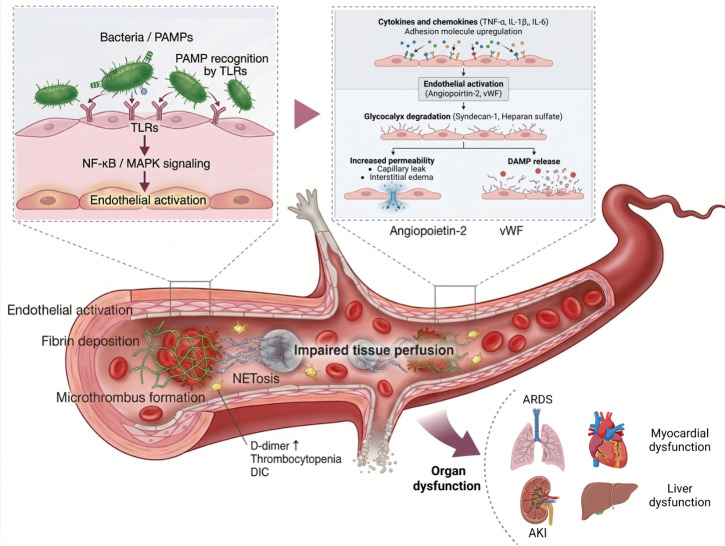
Endothelial activation and microvascular collapse in sepsis. Endothelial activation begins with Toll-like receptor–mediated recognition of pathogen-associated and damage-associated molecular patterns, triggering nuclear factor kappa B and mitogen-activated protein kinase signaling. These pathways promote the release of proinflammatory cytokines, including tumor necrosis factor alpha, interleukin-1 beta, and interleukin-6, and increase endothelial adhesion molecule expression. Recruited neutrophils and macrophages release proteases, matrix metalloproteinases, and reactive oxygen species, which degrade the endothelial glycocalyx and disrupt VE-cadherin-dependent adherens junctions. This increases vascular permeability, promotes interstitial edema, and generates glycocalyx fragments that may act as damage-associated molecular signals, further amplifying inflammation [10,126,127]. The exposed endothelial surface facilitates complement activation, platelet adhesion, fibrin deposition, NETosis, and microthrombus formation, contributing to disseminated intravascular coagulation, impaired capillary perfusion, endothelial cell death, and progression toward multiorgan failure, particularly in organs with high metabolic demands or fenestrated capillary beds, such as the lungs, kidneys, liver, and heart [47,87,119,120,128,129].

Together, these findings support a unifying concept in which sepsis is not merely an inflammatory disorder but a syndrome of systemic endothelial and microvascular failure. The convergence of glycocalyx degradation, junctional disruption, oxidative injury, and microthrombosis transforms the endothelium from a regulator of vascular homeostasis into a driver of organ dysfunction. This paradigm provides the mechanistic basis for understanding why tissue hypoxia may persist despite antimicrobial therapy and hemodynamic correction, and why therapeutic strategies must address preservation of endothelial integrity, restoration of microvascular flow, and interruption of thromboinflammatory feedback loops [47,87,119,120] (Figure 2).

Together, these mechanisms support a systems-level interpretation of sepsis as a syndrome of endothelial and microvascular failure. Diverse upstream triggers converge on a restricted set of endothelial injury processes, including glycocalyx degradation, junctional disassembly, oxidative damage, mitochondrial dysfunction, complement activation, platelet–leukocyte interactions, and NET formation [42,125,130,131,132]. The functional role of these mechanisms is integrative rather than additive: barrier failure amplifies inflammation, inflammation accelerates vascular breakdown, and thromboinflammation obstructs capillary flow. The resulting microcirculatory incoherence is characterized by heterogeneous perfusion, regional hypoxia, and impaired oxygen extraction despite normalized systemic hemodynamics [42]. At this stage, tissue injury becomes increasingly decoupled from pathogen burden and reflects collapse of vascular self-regulation, establishing a mechanistic foundation for strategies aimed at preserving endothelial integrity and preventing irreversible organ dysfunction [36,41,125].

### Microvascular Endothelial Perfusion Collapse

Microcirculatory dysfunction represents one of the most critical pathophysiological mechanisms driving the progression of sepsis toward multiorgan dysfunction and refractory shock. Despite major advances in hemodynamic resuscitation strategies, restoration of macrocirculatory variables such as mean arterial pressure, cardiac output, and systemic oxygen delivery does not guarantee recovery of effective tissue perfusion [94,133,134]. This phenomenon, known as loss of hemodynamic coherence, describes the dissociation between apparent systemic hemodynamic stability and persistent microcirculatory failure, including severe capillary flow heterogeneity, reduced perfused vascular density, impaired tissue oxygen extraction, and cellular bioenergetic collapse [134]. During sepsis, sustained inflammatory activation transforms the microcirculation into a structurally and functionally disorganized vascular network incapable of maintaining homogeneous oxygen and nutrient distribution to metabolically vulnerable tissues [124,135].

The biological basis of this process lies in progressive endothelial dysfunction. Under physiological conditions, the endothelium functions as a dynamic immunometabolic organ regulating vascular tone, capillary permeability, leukocyte trafficking, coagulation balance, and tissue metabolic adaptation through production of nitric oxide, prostacyclin, thrombomodulin, and intracellular antioxidant systems [136,137]. During sepsis, persistent exposure to bacterial endotoxins, pathogen-associated molecular patterns (PAMPs), damage-associated molecular patterns (DAMPs), and proinflammatory cytokines induces sustained activation of Toll-like receptors, NF-κB signaling, inflammasomes, and oxidative stress pathways, promoting endothelial transition toward a proinflammatory, prothrombotic, and vasoplegic phenotype [138,139,140]. This transformation is characterized by progressive glycocalyx degradation, increased expression of adhesion molecules including ICAM-1 and VCAM-1, massive von Willebrand factor release, disruption of intercellular junction integrity, and loss of shear stress-dependent vasomotor autoregulation [19,20].

Glycocalyx degradation constitutes a central event in this pathophysiological transition. Loss of this endothelial surface structure impairs mechanotransduction, vascular tone regulation, and anticoagulant properties of the luminal interface, favoring leukocyte adhesion, platelet activation, and exposure of a highly thrombogenic endothelial surface. Simultaneously, immunothrombotic activation promotes formation of fibrin–platelet microaggregates that increase intraluminal resistance and fragment capillary flow continuity. The combined effects of segmental vasoplegia, immunothrombosis, and vascular leakage generate chaotic redistribution of microvascular perfusion with simultaneous regions of hyperflow, flow stagnation, and complete capillary nonperfusion [15,19].

Clinically, these alterations translate into persistent hyperlactatemia, impaired tissue oxygen extraction, septic encephalopathy, acute kidney injury, and progression toward multiorgan failure [133,141]. The lungs, kidneys, liver, and myocardium are particularly vulnerable due to their elevated metabolic demands and dependence on homogeneous capillary perfusion. In the lung, endothelial injury promotes alveolocapillary permeability and acute respiratory distress syndrome. In the kidney, cortical and medullary microvascular heterogeneity contributes to persistent tubular hypoxia and acute kidney injury. At the myocardial level, microcirculatory dysfunction promotes cardiomyocyte mitochondrial impairment and septic myocardial depression [142,143].

The incorporation of bedside microcirculatory assessment technologies has enabled translation of this pathophysiological understanding into critical care practice. Techniques such as sidestream dark field and incident dark field sublingual videomicroscopy allow for direct evaluation of functional capillary density, proportion of perfused vessels, flow heterogeneity, and glycocalyx structural integrity in real time [144,145]. Clinical studies have demonstrated that persistent microcirculatory abnormalities during septic resuscitation correlate more closely with mortality and organ dysfunction than multiple conventional systemic hemodynamic parameters [133,146]. These observations reinforce the need for resuscitation strategies focused not only on macrocirculatory restoration but also on preservation of endothelial integrity and recovery of effective tissue perfusion [141].

Simultaneously, multiple forms of critical illness converge toward a shared phenotype of acute endotheliopathy characterized by vascular hyperpermeability, immunothrombosis, mitochondrial dysfunction, and progressive microvascular perfusion collapse [2]. Both sepsis and severe trauma induce transition toward a proinflammatory and prothrombotic endothelial state; however, their initiating mechanisms differ substantially [147]. Sepsis is predominantly driven by PAMP-mediated signaling, persistent cytokine amplification, and sustained immunothrombotic activation, whereas traumatic endotheliopathy is primarily associated with massive DAMP release, hemorrhagic hypoperfusion, direct endothelial mechanical injury, and catecholamine storm physiology [148]. Despite these etiological differences, both syndromes converge upon common terminal pathways of endothelial dysfunction, microvascular disorganization, and tissue perfusion failure [149]. This comparison supports the concept that endothelial failure represents a shared terminal pathway across critical illness, while pathogen-driven signaling in sepsis and hemorrhagic-mechanical injury in trauma define distinct upstream therapeutic priorities.

Perfusion failure represents the terminal consequence of the interaction between systemic inflammation, immunothrombosis, and progressive microvascular collapse. Inability to sustain effective oxygen exchange induces persistent cellular hypoxia, metabolic transition toward anaerobic glycolysis, and progressive lactate accumulation [141,149]. At the molecular level, reduced physiological nitric oxide bioavailability, excessive reactive oxygen species production, altered mitochondrial dynamics, and bioenergetic failure perpetuate irreversible cellular dysfunction [145,150]. Clinically, this process culminates in refractory oliguria, persistent hypoxemia, neurological deterioration, tissue necrosis, and irreversible septic shock [147,150]. This pathophysiological convergence positions endothelial collapse as one of the central biological mechanisms underlying critical illness, establishing the endothelium as a priority target for advanced monitoring and endothelial-directed therapeutic strategies [135,136].

## 9. Organ-Specific Endothelial Vulnerability

Endothelial injury in sepsis is not uniformly distributed across organs. Although systemic inflammation and thromboinflammation provide common upstream triggers, the vascular response varies according to local endothelial architecture, capillary permeability, metabolic demand, and pathogen-specific virulence patterns. This organ-level heterogeneity helps explain why hepatic, renal, pulmonary, and cardiac dysfunction may progress at different rates despite comparable systemic inflammatory profiles [13,31,68,121,122,123].

Organs with fenestrated or highly permeable capillary beds, such as the liver and kidneys, are particularly susceptible to early endothelial disruption. Similarly, organs with high metabolic requirements, such as the heart and lungs, are vulnerable to small reductions in microvascular oxygen delivery [31,121,122,123]. In contrast, tissues protected by continuous endothelial barriers and tighter junctional architecture, including the brain, may exhibit relatively delayed involvement, although this protection can be overcome during sustained systemic injury [34,68].

Pathogen-specific mechanisms may further shape these organ-level vascular responses. Sepsis caused by highly virulent pathogens, such as methicillin-resistant *Staphylococcus aureus*, illustrates how microbial programs can imprint distinct endothelial and proteomic patterns across vascular beds [13,68]. In this setting, endothelial remodeling may precede overt organ dysfunction and reflect organotypic vascular responses rather than uniform systemic inflammation [34,68]. *S. aureus*-associated endothelial invasion, mediated in part by fibronectin-binding proteins and integrin clustering, may favor intracellular persistence and focal vascular injury, whereas Gram-negative pathogens more commonly induce diffuse endothelial activation through lipopolysaccharide-driven innate immune signaling [37,83,93].

In the liver, the acute-phase response increases the synthesis of haptoglobin, orosomucoid, and complement components, while complement deposition on injured endothelium contributes to microvascular obstruction and septic coagulopathy [31,121,122,123]. Septic vascular remodeling may also involve overexpression of lubricin within hepatic sinusoids, potentially representing an endogenous attempt to restore endothelial lubrication and barrier function. However, accumulation of lubricin within microvascular aggregates, particularly in the presence of circulating hyaluronic acid, may paradoxically enhance leukocyte adhesion and local coagulation, aggravating sinusoidal dysfunction and tissue hypoxia [31,121,122,123].

Pulmonary endothelial injury is especially clinically relevant because disruption of the alveolar–capillary barrier directly impairs gas exchange. Loss of endothelial integrity allows protein-rich exudate to enter the alveolar space, diluting surfactant, reducing lung compliance, and producing refractory hypoxemia. Endothelial activation also promotes von Willebrand factor release, neutrophil recruitment, reactive oxygen species generation, basement membrane injury, and microvascular thrombosis, all of which contribute to acute lung injury and acute respiratory distress syndrome [151,152,153]. The resulting imbalance between oxidative-inflammatory injury and regenerative metabolic capacity determines whether the pulmonary endothelium recovers or progresses toward sustained barrier failure.

Renal and cardiac involvement also reflect organ-specific endothelial vulnerability. In the kidney, microvascular dysfunction, endothelial swelling, calcium-dependent signaling, and capillary obstruction impair glomerular filtration and contribute to septic acute kidney injury [37,83,93]. In the heart, endothelial dysfunction and microvascular thrombosis compromise coronary perfusion and contribute to septic cardiomyopathy [37,83,93]. Across these organs, the common endpoint is loss of coordinated perfusion, impaired oxygen extraction, and propagation of local inflammation.

At the molecular level, several pathways recur across organ systems. Disruption of the angiopoietin–Tie2 axis promotes junctional instability and vascular leakage, while activation of RhoA, myosin light-chain kinase, and oxidative stress pathways destabilizes cytoskeletal organization and endothelial cohesion. Concurrent suppression of endogenous anticoagulant mechanisms, including protein C, protein S, and tissue factor pathway inhibitor, facilitates thrombin generation, fibrin deposition, and microthrombus formation [37,83,93,94,95]. These shared mechanisms explain why distinct organs may display different clinical patterns while converging on a common endpoint of microvascular failure.

Endothelial repair capacity also differs by organ and may influence recovery. In acute lung injury and acute respiratory distress syndrome, endothelial progenitor cells are mobilized from the bone marrow and may contribute to vascular repair through migration, adhesion, proliferation, and release of trophic factors such as VEGF and angiopoietins [151,153]. This reparative process depends on adequate metabolic substrate availability, mitochondrial function, and controlled nitric oxide signaling. Excess oxidative stress, however, can convert nitric oxide into peroxynitrite, amplifying cellular injury rather than restoring vascular tone [106,151,152,154]. Early measurement of endothelial progenitor cell colony-forming units has been proposed as an indirect marker of regenerative endothelial capacity, with higher counts associated with better survival in some cohorts [151,152].

These observations indicate that sepsis should be understood not only as systemic endothelial failure but also as an organ-patterned vascular disorder. The same core mechanisms—glycocalyx degradation, junctional disruption, oxidative injury, complement activation, and microthrombosis—manifest differently depending on local vascular structure, metabolic demand, and pathogen-specific injury programs. Recognizing this heterogeneity is essential for interpreting organ dysfunction, refining endothelial biomarkers, and designing targeted strategies aimed at preserving microvascular function in vulnerable vascular beds.

## 10. Mechanistic Biomarkers of Endothelial Injury

Endothelial biomarkers in sepsis should be interpreted not merely as isolated indicators of vascular damage, but as mechanistic readouts of specific transitions within the immunovascular network. Glycocalyx disruption, junctional destabilization, loss of anticoagulant signaling, thromboinflammatory activation, and impaired tissue perfusion generate distinct circulating signals. However, only a subset of biomarkers provides coherent information about both structural injury and functional consequence [89,155].

Among these, syndecan-1 and soluble thrombomodulin are particularly relevant because they represent complementary dimensions of endothelial failure. Syndecan-1 is a core proteoglycan of the endothelial glycocalyx and serves as a surrogate marker of glycocalyx shedding. Its release into the circulation reflects early structural loss of the endothelial surface layer and is linked to increased permeability, altered mechanotransduction, and impaired vascular barrier function [156,157]. In sepsis, pathogen- and damage-associated molecular patterns, oxidative stress, matrix metalloproteinases, heparanases, and hyaluronidases contribute to glycocalyx cleavage and syndecan-1 release [21,22,24].

Soluble thrombomodulin reflects a different but complementary axis of endothelial injury. Thrombomodulin is a membrane-bound glycoprotein with anticoagulant and cytoprotective functions. Its shedding during endothelial damage indicates loss of anticoagulant signaling and transition toward a procoagulant endothelial phenotype [21,88,157,158]. Together, syndecan-1 and soluble thrombomodulin capture two central dimensions of septic endothelial failure: disruption of barrier integrity and impairment of anticoagulant homeostasis.

This pairing is clinically and mechanistically meaningful because these biomarkers map the transition from regulated endothelial homeostasis to systemic microvascular instability. Elevated levels of syndecan-1 and soluble thrombomodulin have been associated with progressive organ dysfunction, septic shock, higher lactate concentrations, disseminated intravascular coagulation, and increased mortality [21,22,24,159]. Their correlation with clinical severity scores, including Sequential Organ Failure Assessment and National Early Warning Score, supports their potential value as markers of disease trajectory rather than merely static injury signals [21,157].

Other endothelial and contextual biomarkers provide additional information about specific components of the septic vascular phenotype. Angiopoietin-2 reflects disruption of the Tie2 axis and junctional instability, linking endothelial activation to permeability, vascular leak, and acute respiratory distress syndrome. VEGF may act as a context-dependent amplifier of permeability and repair signaling, although its association with outcomes appears heterogeneous. Plasminogen activator inhibitor-1 reflects impaired fibrinolysis and persistent microthrombosis, whereas von Willebrand factor indicates endothelial activation and platelet adhesion. Within sepsis-associated DIC, PAI-1 and vWF are particularly relevant because they connect endothelial activation with antifibrinolytic failure, platelet adhesion, fibrin persistence, and microvascular thrombus formation. In contrast, biomarkers such as interleukin-6, high-mobility group box 1, and N-terminal pro-B-type natriuretic peptide provide information about systemic inflammation, damage-associated signaling, or myocardial stress, but are less specific for endothelial injury itself.

The major limitation of these biomarkers is that most evidence remains observational, with substantial heterogeneity in timing of sampling, assay platforms, patient phenotypes, infection sources, and clinical endpoints. Therefore, endothelial biomarkers should not yet be interpreted as stand-alone diagnostic or therapeutic triggers. Their current value lies in mechanistic phenotyping, prognostic enrichment, and hypothesis generation for biomarker-guided strategies. Future studies should define standardized thresholds, determine temporal trajectories during early sepsis, and evaluate whether combining endothelial biomarkers with clinical scores improves prediction of organ failure, shock progression, and mortality [14,21,88,124,160] (Table 1).

## 11. Experimental Models and Therapeutic Implications

Experimental models are essential for understanding how endothelial dysfunction develops, persists, and becomes therapeutically targetable in sepsis. No single model fully reproduces the biological heterogeneity of human sepsis; however, complementary systems allow specific dimensions of immunovascular failure to be dissected, including early endothelial activation, impaired barrier repair, maladaptive immune reprogramming, and microvascular collapse [161,162,163,164,165,166].

### 11.1. Experimental Platforms for Endothelial Dysfunction and Microvascular Failure

Advanced in vitro platforms provide mechanistic validation that septic vascular injury is not only biochemical but also mechanically driven [167,168,169]. Organ-on-chip systems and vascular bioreactors reproduce human endothelial monolayers under controlled shear stress, hydrostatic pressure, and cyclic stretch, allowing for real-time assessment of permeability, junctional stability, and barrier integrity [170,171,172]. These models show that multiaxial mechanical loading, particularly equibiaxial strain, can disrupt endothelial monolayers and increase permeability in a manner predicted by strain energy density [67,172].

### 11.2. Animal, Endotoxin, Two-Hit, and Comorbidity-Based Models

Animal and endotoxin-based models capture different stages of septic host-response failure. One-hit models, typically induced by lipopolysaccharide exposure or cecal ligation and puncture, reproduce early innate immune activation, glycocalyx degradation, adhesion molecule expression, impaired mechanotransduction, and initial permeability changes [161,164]. Two-hit models, in which an initial insult is followed by a secondary infectious challenge, reproduce failure of resolution and impaired immune responsiveness, with persistent junctional instability, compromised anticoagulant signaling, leukocyte adhesion, and microthrombotic activity [162,173,174,175]. Endotoxin tolerance models demonstrate maladaptive immune reprogramming toward a hyporesponsive state, characterized by reduced cytokine responsiveness, impaired nitric oxide production, diminished vasoregulatory capacity, and a rigid microvascular phenotype unable to adapt to hemodynamic fluctuations. Finally, models incorporating aging, comorbidities, or altered microbiota better reproduce baseline endothelial vulnerability, including glycocalyx thinning, chronic oxidative stress, altered intracellular signaling, and lower thresholds for barrier disruption [91,163,165].

Together, these models suggest that endothelial dysfunction follows a trajectory from reversible activation to persistent injury and ultimately to structural and functional breakdown. At the vascular level, this trajectory includes loss of flow sensing, progressive permeability, reduced nitric oxide bioavailability, impaired anticoagulant signaling, and microcirculatory incoherence [91,127,161,163,165]. Clinically, this aligns with persistent tissue hypoxia, secondary infections, and organ dysfunction despite apparent stabilization of systemic hemodynamics [96,175]. These models therefore show that sepsis is not a linear inflammatory process but a dynamic systems-level failure in which endothelial dysfunction links experimental observations to clinical outcomes [91,163,165].

### 11.3. Endothelium-Friendly Supportive Care and Microvascular Protection

Several experimental interventions have emerged from this mechanistic framework. Hemodynamic strategies can be interpreted as endothelial-modulating interventions when they reduce mechanical stress and preserve microvascular coherence. Carefully titrated vasopressors may restore vascular tone while avoiding excessive shear stress, and fluid administration guided by dynamic indices may limit capillary hydrostatic overload, glycocalyx disruption, and vascular leakage [176,177,178,179,180]. Optimization of cardiac output may support microvascular flow, while lactate clearance provides an indirect marker of improved tissue perfusion [128,181]. In mechanically ventilated patients, limiting plateau pressure and adjusting positive end-expiratory pressure may reduce transmission of injurious intrathoracic forces to the pulmonary microcirculation [182,183]. These strategies are not endothelial-specific therapies, but they may reduce endothelial strain and limit progression from barrier dysfunction to microvascular collapse [4,184].

### 11.4. Molecular, Regenerative, and Signaling-Targeted Approaches

Molecular and regenerative approaches remain largely preclinical. miR-126 regulates endothelial integrity, angiogenic signaling, vascular permeability, inflammation, and repair pathways in experimental sepsis models [12,185]. Although protective effects on endothelial stability have been reported, its therapeutic role remains insufficiently validated and is limited by context-dependent expression, systemic effects, and uncertain delivery strategies [12,186,187]. Similarly, EPC-derived exosomes have shown promising effects in murine cecal ligation and puncture models. By delivering miR-126-3p and miR-126-5p, these vesicles reduce VCAM-1 and HMGB1 expression, attenuate inflammatory cytokine release, decrease vascular leakage, and improve organ injury markers in preclinical settings [188,189,190]. However, variability in exosome cargo, biodistribution, targeting specificity, and manufacturing standardization remains a major barrier to clinical translation.

Other pathways, including TRP channel signaling and VEGF-mediated repair responses, should be interpreted as context-dependent modulators rather than universal therapeutic targets. TRPV4 activation promotes calcium influx, cytoskeletal contraction, junctional opening, and capillary leakage, while TRPM7 links oxidative stress, ionic imbalance, mitochondrial dysfunction, and impaired endothelial repair [25,99,191,192,193]. VEGF signaling may support endothelial survival and repair during early injury but can amplify permeability when sustained or excessive [193,194,195,196,197,198]. Therefore, interventions targeting these pathways require careful attention to timing, disease stage, and organ-specific vascular responses.

### 11.5. Endothelial Immunothrombotic and Immunomodulatory Therapeutic Strategies

Beyond supportive and regenerative approaches, several therapeutic strategies directly target the endothelial–immunothrombotic interface in sepsis-associated coagulopathy and DIC. These include recombinant human soluble thrombomodulin, heparin-based anticoagulant strategies, defibrotide-mediated endothelial stabilization, and selected immunomodulatory interventions such as IL-6 blockade in hyperinflammatory phenotypes.

#### 11.5.1. Recombinant Human Soluble Thrombomodulin (rhTM)

Recombinant human soluble thrombomodulin (rhTM) represents one of the most sophisticated translational attempts to therapeutically modulate the endothelial–immunothrombotic interface in sepsis-associated disseminated intravascular coagulation (DIC) [35,199,200]. Rather than functioning as a conventional anticoagulant centered exclusively on coagulation cascade suppression, rhTM was developed as a biologically integrated endothelial regulator capable of simultaneously attenuating thrombin-mediated coagulation, endothelial inflammatory activation, and microvascular dysfunction [199,200]. Derived from the extracellular domain of native endothelial thrombomodulin, rhTM restores part of the endogenous anticoagulant architecture lost during septic endothelial injury by binding circulating thrombin and promoting protein C activation, thereby limiting amplification of intravascular coagulation and downstream thromboinflammatory propagation [200,201].

Its relevance extends beyond coagulation control. Experimental and translational studies suggest that rhTM exerts direct endothelial protective effects through attenuation of leukocyte–endothelium interaction, reduction of inflammatory cytokine amplification, stabilization of microvascular perfusion, and preservation of endothelial barrier integrity. This is particularly important in sepsis, where endothelial collapse progressively transforms the microcirculation into a rigid, hypoperfused, and metabolically dysfunctional vascular network incapable of sustaining homogeneous oxygen delivery. Within this framework, rhTM emerged in Japan not merely as a hemostatic intervention, but as a therapeutic strategy targeting the vascular biology of septic organ dysfunction itself [199,200].

Although large randomized analyses, including the SCARLET trial and subsequent meta-analytical evaluations, have demonstrated heterogeneous effects on mortality reduction, the mechanistic significance of rhTM remains substantial because it directly addresses the convergence between inflammation, coagulation, endothelial activation, and microvascular failure. Importantly, Japan became the first country to incorporate rhTM into clinical practice for sepsis-associated DIC following phase III clinical evaluation, establishing a therapeutic precedent centered on endothelial-directed immunothrombotic modulation. This paradigm reinforces the contemporary interpretation of septic DIC as a dynamic endothelial immunovascular syndrome rather than an isolated coagulation disorder, highlighting the endothelium as both a mechanistic epicenter and a therapeutic target in sepsis-induced multiorgan failure [74,199,200].

#### 11.5.2. Heparin-Mediated Endothelial Protection in Sepsis-Induced Coagulopathy

Unfractionated heparin (UFH) and low-molecular-weight heparins (LMWH) have gained increasing relevance as immunothrombotic modulation strategies in sepsis, particularly in patients with sepsis-induced coagulopathy (SIC) and disseminated intravascular coagulation (DIC) [202,203]. Beyond their classical anticoagulant activity mediated through antithrombin III, emerging clinical evidence suggests that these agents may directly interfere with central mechanisms of endothelial injury, microvascular activation, and systemic perfusion dysfunction. A large multicenter Japanese observational study involving more than 30,000 septic patients demonstrated that early UFH administration within the first 72 h of hospitalization was associated with a significant reduction in in-hospital mortality, especially among patients with moderate SIC and SIC scores ranging from 3 to 4 [202,203,204]. Importantly, this survival benefit was not accompanied by a significant increase in major bleeding complications, suggesting that early anticoagulant intervention may be most effective during the initial phases of immunothrombotic activation before the establishment of advanced microvascular rigidity and severe endothelial failure [202,204].

In parallel, meta-analytical evaluations of LMWH have demonstrated reductions in 28-day mortality, lower APACHE II scores, and improvement in coagulation parameters, including prothrombin time and platelet counts [203,204]. These findings reinforce the concept that targeted anticoagulant therapy may modulate the pathophysiological progression of septic coagulopathy and limit amplification of capillary microthrombosis [202,203,204].

More recently, mechanistic investigations have identified direct endothelial protective properties of UFH independent of its conventional anticoagulant activity, including inhibition of Drp1-mediated mitochondrial translocation, preservation of endothelial mitochondrial homeostasis, reduction of vascular leakage, and stabilization of microcirculatory integrity. Collectively, these observations position UFH and LMWH as potential endothelial-protective therapies directed against immunothrombosis and the progressive perfusion collapse characteristic of severe sepsis [202,204].

#### 11.5.3. Defibrotide Endothelial Stabilization Therapy

Defibrotide has emerged as a promising endothelial-directed therapeutic candidate in sepsis due to its capacity to modulate multiple pathways involved in endothelial activation, immunothrombosis, and microvascular dysfunction. Unlike conventional anticoagulants focused primarily on coagulation cascade inhibition, defibrotide exerts pleiotropic endothelial-protective effects that directly target the proinflammatory and prothrombotic phenotype characteristic of septic endothelial injury [205,206]. Experimental studies using endothelial cells exposed to sera from patients with sepsis, severe sepsis, and septic shock demonstrated progressive endothelial activation proportional to disease severity, characterized by increased expression of adhesion molecules including ICAM-1 and VCAM-1, augmented von Willebrand factor (vWF) release, enhanced extracellular matrix thrombogenicity, platelet adhesion, and activation of intracellular inflammatory signaling pathways such as p38 MAPK. Defibrotide significantly attenuated these alterations, reducing endothelial inflammatory activation, limiting thrombogenic endothelial remodeling, and preserving endothelial functional integrity under septic conditions [205,207].

Mechanistically, defibrotide interacts directly with endothelial cell membranes and undergoes intracellular internalization, promoting anti-inflammatory, antithrombotic, profibrinolytic, antiapoptotic, and angio-protective responses [207,208]. These effects are particularly relevant in sepsis, where sustained endothelial hyperactivation contributes to glycocalyx disruption, capillary leakage, microvascular thrombosis, tissue hypoperfusion, and multiorgan dysfunction. Importantly, experimental evidence suggests that defibrotide may preserve endothelial barrier stability and reduce endothelial–platelet interaction even during advanced septic inflammatory states. Collectively, these findings position defibrotide as a potential endothelial recalibration strategy directed against septic immunovascular collapse. Its incorporation into endothelial-centered therapeutic models reinforces the concept that modulation of endothelial biology may represent a critical intervention point for limiting progression toward irreversible microcirculatory failure and multiorgan dysfunction in severe sepsis [207,208].

#### 11.5.4. IL-6 Blockade and Precision Immunomodulation in Sepsis-Associated DIC

IL-6 blockade with tocilizumab has emerged as a potential precision immunomodulatory strategy in selected septic patients with sepsis-associated DIC or sepsis-induced coagulopathy characterized by hyperinflammatory phenotypes, cytokine storm physiology, pancytopenia, and dysregulated IL-6 signaling. Although not routinely recommended for standard sepsis management, accumulating translational evidence suggests that excessive IL-6 amplification contributes directly to endothelial activation, vascular permeability, immunothrombotic dysregulation, mitochondrial dysfunction, and progression toward multiorgan failure. Tocilizumab, a humanized monoclonal antibody targeting both soluble and membrane-bound IL-6 receptors, suppresses IL-6-mediated inflammatory signaling through inhibition of the IL-6/IL-6R/gp130 pathway, thereby attenuating cytokine propagation and endothelial inflammatory injury [209,210].

Clinical observations in critically ill septic patients presenting with pancytopenia and hyperinflammatory syndromes have suggested potential hemodynamic stabilization and reduction in inflammatory burden following tocilizumab administration, particularly in patients exhibiting severe cytokine-driven immune dysregulation [209,210,211]. Mechanistically, IL-6 blockade may reduce endothelial hyperactivation, decrease expression of adhesion molecules, limit leukocyte–endothelium interaction, attenuate capillary leakage, and suppress secondary amplification of immunothrombotic pathways. Furthermore, data derived from severe inflammatory syndromes, including cytokine storm models, suggest that post-treatment IL-6 dynamics may correlate with inflammatory control and clinical trajectory following tocilizumab exposure [209,211]. However, the use of tocilizumab in sepsis remains biologically complex because IL-6 also plays a critical role in antimicrobial host defense and immune coordination. Consequently, IL-6 inhibition should be interpreted as a targeted immunomodulatory intervention potentially applicable to carefully selected hyperinflammatory septic phenotypes rather than as a universal therapeutic strategy for sepsis-associated immune dysfunction [209,210,211].

The broader therapeutic implication is that endothelial protection in sepsis will likely require combination strategies rather than single-pathway interventions. Potential approaches include preservation of the glycocalyx, stabilization of intercellular junctions, attenuation of thromboinflammation, modulation of oxidative stress, enhancement of mitochondrial repair, optimization of microvascular flow, and phenotype-specific immunomodulation. Yet most candidate therapies remain supported primarily by preclinical, observational, or context-specific clinical evidence. Translation will require standardized endothelial biomarkers, validated timing windows, phenotype-specific patient selection, and trials designed to test whether preserving endothelial coherence improves organ function and survival [35,74,199,200,201,202,203,204,205,206,207,208,209,210,211] (Table 2).

Overall, experimental models have shifted the understanding of sepsis from a linear inflammatory disorder toward viewing it as a dynamic immunovascular process. They show that endothelial dysfunction can be initiated by inflammatory signals, amplified by mechanical and metabolic stress, sustained by thromboinflammation, and only partially reversed by current supportive care. This provides a rational foundation for future therapies aimed not simply at suppressing inflammation, but at restoring endothelial barrier function, microvascular coherence, and host vascular resilience.

## 12. Discussion

Endothelial dysfunction represents a critical turning point in the progression from localized infection to systemic microvascular failure and multiorgan dysfunction. The evidence reviewed here supports a model in which glycocalyx degradation, junctional destabilization, oxidative injury, mitochondrial dysfunction, thromboinflammation, and impaired repair converge to disrupt microvascular coherence [1,21,22]. In this framework, sepsis is not only an inflammatory or infectious syndrome, but a dynamic disorder of vascular homeostasis in which the endothelium becomes both a target and an amplifier of host-derived injury.

A central implication of this model is that endothelial injury helps explain the dissociation between pathogen control, systemic hemodynamic stabilization, and persistent tissue hypoxia. Activation of Toll-like receptor signaling by pathogen- and damage-associated molecular patterns triggers nuclear factor kappa B and mitogen-activated protein kinase pathways, cytokine release, reactive oxygen species generation, and matrix metalloproteinase activation [47,106,188]. These processes degrade the glycocalyx, disrupt adherens junctions, expose the endothelial surface to leukocytes and platelets, and promote complement activation and microthrombus formation. The resulting cycle of barrier breakdown, coagulation activation, vascular leakage, and impaired perfusion can continue even after the initial infectious trigger is controlled [47,106,188].

The clinical expression of this process varies by organ. In the lungs, alveolar–capillary leakage contributes to acute respiratory distress syndrome and refractory hypoxemia. In the kidneys, endothelial injury, calcium-dependent signaling, and microvascular obstruction impair glomerular filtration and contribute to septic acute kidney injury. In the liver, sinusoidal endothelial dysfunction promotes leakage of albumin and inflammatory mediators, hepatocellular stress, and local immune amplification. In the heart, altered nitric oxide and endothelin signaling, together with microvascular thrombosis, contribute to septic cardiomyopathy. Organs with tighter endothelial barriers, such as the brain, may be relatively protected early in the disease course, but this protection can be overcome when systemic endothelial injury persists [47,106,188].

Mechanistic biomarkers provide a potential bridge between this pathophysiological framework and clinical interpretation. Syndecan-1 and soluble thrombomodulin are particularly relevant because they reflect complementary dimensions of endothelial failure: glycocalyx shedding and loss of endothelial anticoagulant/cytoprotective signaling. Their association with organ dysfunction, lactate elevation, septic shock, and mortality supports their value as indicators of disease trajectory rather than isolated markers of injury [1,21,22]. However, their clinical use remains limited by observational evidence, small sample sizes, heterogeneous cohorts, variable sampling times, non-standardized assays, and insufficiently validated thresholds.

These limitations define several priorities for future research. First, studies should establish standardized measurement protocols and clinically meaningful cutoffs for endothelial biomarkers across different sepsis phenotypes. Second, longitudinal sampling is needed to clarify the temporal sequence of glycocalyx shedding, thrombomodulin release, barrier recovery, and organ dysfunction during the first days of critical illness. Third, endothelial biomarkers should be tested in combination with clinical scores, lactate kinetics, coagulation markers, and microcirculatory monitoring to determine whether they improve risk stratification beyond conventional tools. Finally, interventional trials are needed to determine whether endothelial-guided strategies can modify outcomes rather than merely identify high-risk patients [1,21,22].

Therapeutically, this review supports a cautious but biologically plausible shift toward endothelial-preserving strategies. Potential approaches include limiting glycocalyx degradation, stabilizing intercellular junctions, reducing oxidative stress, modulating microvascular coagulopathy, and tailoring fluid and vasopressor therapy to avoid worsening vascular leakage.

As discussed above, strategies such as recombinant human soluble thrombomodulin, heparin-based anticoagulant therapy, defibrotide, and IL-6 blockade illustrate different levels of endothelial-directed intervention, ranging from immunothrombotic modulation to phenotype-specific immunomodulation.

However, most endothelial-targeted interventions remain experimental, supported by indirect clinical evidence, or applicable only to selected phenotypes [47,106,188]. This includes heparanase inhibitors, antioxidants, membrane-stabilizing agents, exosome-based therapies, regenerative approaches, endothelial-directed anticoagulant strategies, and precision immunomodulatory interventions. Therefore, endothelial restoration should currently be viewed as a mechanistic and translational objective rather than an established universal therapeutic standard.

Overall, endothelial dysfunction reframes sepsis as a progressive disorder of microvascular regulation. By linking inflammation, oxidative stress, coagulation, barrier failure, and impaired oxygen delivery, this model explains why organ dysfunction may persist despite antimicrobial therapy and apparent hemodynamic correction. The next step is to translate this pathophysiological understanding into standardized biomarker platforms, validated experimental models, and clinical trials designed to test whether preservation of endothelial coherence can improve recovery and survival in sepsis.

## 13. Conclusions

Sepsis can be understood as a condition in which failure to restore endothelial coherence sustains organ dysfunction beyond the initial infectious trigger. Once the vascular interface loses its capacity to integrate inflammatory, mechanical, metabolic, and coagulative signals, the host response may shift from adaptive defense to self-sustaining microvascular instability. This transition is driven by the convergence of glycocalyx degradation, junctional disruption, oxidative injury, mitochondrial dysfunction, thromboinflammation, and impaired endothelial repair.

Mechanistically, this state is maintained through feedback loops linking permeability, coagulation, inflammation, and tissue hypoxia. The consequence is not simply vascular leakage or microthrombosis in isolation, but the emergence of a disordered microcirculatory network unable to sustain coordinated oxygen delivery. This framework explains why tissue hypoxia and organ dysfunction may persist despite pathogen control and apparent systemic hemodynamic stabilization.

Within this model, endothelial biomarkers should be interpreted as indicators of vascular system state rather than isolated markers of injury. Likewise, therapeutic strategies should move beyond single-pathway modulation toward approaches that preserve or restore endothelial barrier function, microvascular flow, and vascular resilience. Current evidence does not support any single intervention as sufficient to reverse established endothelial failure, underscoring the need for early recognition, standardized biomarker assessment, and phenotype-guided therapeutic development.

Overall, sepsis is not resolved by eliminating the pathogen alone, but by restoring the host’s capacity to maintain vascular coherence under stress. This endothelial-centered perspective provides a pathophysiological foundation for future precision medicine strategies focused on dynamic monitoring, individualized host-response modulation, and preservation of microvascular integrity as a determinant of recovery.

## Figures and Tables

**Table 1:** Biomarkers linked to endothelial injury and microvascular dysfunction in sepsis. Direct endothelial and thromboinflammatory biomarkers include syndecan-1, soluble thrombomodulin, angiopoietin-2, von Willebrand factor, and plasminogen activator inhibitor-1. Contextual biomarkers such as interleukin-6, high-mobility group box 1, and N-terminal pro-B-type natriuretic peptide reflect systemic inflammation, tissue damage signaling, or end-organ stress rather than endothelial injury per se.

Domain	Biomarker	What It Reflects (Mechanism)	Typical Clinical Association	Evidence (Type)	Common Endpoints
Glycocalyx/structural injury	Syndecan-1	Glycocalyx shedding (proteoglycan release)	Higher severity, hypoperfusion, ↑ SOFA, higher mortality	ICU observational cohorts	Mortality; SOFA; lactate; shock
Endothelium/anticoagulant axis	Soluble thrombomodulin (sTM)	Endothelial injury + loss of anticoagulant/cytoprotective signaling	Higher severity, coagulopathy, higher mortality	Observational cohorts	Mortality; DIC; SOFA
Endothelial activation/permeability	Angiopoietin-2 (Ang-2)	Junctional instability via Tie2 antagonism	Higher severity, vascular leak, ARDS, higher mortality	Human cohorts	ARDS; mortality; SOFA
Permeability/repair signaling	VEGF	Permissive amplifier of permeability; adherens junction destabilization	Worse outcomes in selected phenotypes; heterogeneous signals	Observational + preclinical	ARDS; vascular leak; mortality
Thromboinflammation/fibrinolysis	PAI-1	Antifibrinolysis → persistent microthrombosis	Coagulopathy, organ dysfunction, higher mortality	Observational	DIC; mortality
Endothelial activation/platelet adhesion	von Willebrand factor (vWF)	Weibel–Palade body exocytosis; endothelial activation; platelet tethering	Coagulopathy, microvascular thrombosis, DIC, higher severity	Observational and translational studies	DIC; organ dysfunction; mortality
Systemic inflammation	IL-6	Magnitude of inflammatory response	Higher risk in septic shock; severity marker	Observational	Mortality; shock
DAMP/immune amplification	HMGB1	Tissue damage signaling; inflammatory amplification	Heterogeneous associations	Observational	Variable mortality endpoints
Hemodynamic stress	NT-proBNP	Myocardial stress/strain in critical illness	In-hospital mortality; cardiovascular dysfunction	Observational	Mortality; cardiovascular dysfunction

↑ indicates increased or elevated levels/values of the corresponding parameter. **Abbreviations:** ARDS, acute respiratory distress syndrome; DIC, disseminated intravascular coagulation; SOFA, Sequential Organ Failure Assessment.

**Table 2:** Endothelium-, microvasculature-, and immunothrombotic-targeted strategies in sepsis.

Category/Target	Agent	Mechanism	Intended Effect	Evidence	Primary Endpoints
“Endothelium-friendly” supportive care (implementable)	Avoid fluid overload	↓ANP-driven glycocalyx shedding (pathophysiologic rationale)	Less leak/edema; improved microvascular coherence	Clinical (resuscitation strategies; heterogeneous)	Fluid balance; pulmonary edema; ventilation duration; mortality
	Vasopressin as adjunct	Catecholamine-sparing; vascular tone support	Maintain perfusion with less vasopressor-related harm	Clinical (guidelines/ICU practice)	NE dose; MAP; lactate
	Moderate glycemic control	↓ROS/RAGE/NET-related endothelial injury	Indirect glycocalyx/barrier protection	Clinical (indirect)	Hypoglycemia; ICU outcomes; organ dysfunction
Glycocalyx protection	Antithrombin	Binds heparan sulfate + anticoagulant effects	Stabilize glycocalyx; mitigate coagulopathy	Preclinical + mixed clinical	Glycocalyx markers; DIC; mortality
	Albumin/FFP	S1P + protease inhibitors	Barrier support; endothelial protection	Mixed trials	Mortality; shock; vascular leak
Barrier stabilization (S1P axis)	S1P1 agonist (SEW2871)	S1P1 activation → Rac/Akt/ERK → junction stabilization	↓Permeability	Preclinical	Evans blue leakage; edema; survival (models)
Anti-heparanase/glycocalyx preservation	Heparanase inhibitors	↓Heparan sulfate degradation	Preserve glycocalyx	Preclinical	Syndecan-1; vascular leak; SOFA (clinical translation)
GAG remodeling (proposed/heterogeneous)	Sulodexide	GAG mixture; anti-heparanase/anti-inflammatory (model-dependent)	Glycocalyx remodeling	Proposed/limited direct sepsis data	Permeability; glycocalyx markers
Acellular regenerative therapy	EPC-derived exosomes (miR-126-3p/5p)	miR-126 delivery → ↓VCAM-1/↓HMGB1	Restore barrier; ↓leak	Preclinical (murine CLP)	Survival; Evans blue; AST/ALT/BUN
Endothelial stress responses	Rapamycin (autophagy)	↑Endothelial autophagy	Attenuate local barrier injury	Preclinical (AKI-focused)	sTM; VE-cadherin; histology; renal function
Pulmonary endothelial inflammation	MMI-0100 (MK2 inhibitor)	Inhibits MK2 (p38 pathway) → ↓inflammation	Reduce lung endothelial injury	Preclinical	Permeability; inflammation; histology
Endothelin pathway	Tezosentan/Bosentan	ETA/ETB antagonism	Reported microvascular perfusion benefit	Animal/experimental	Regional flow; renal function; survival
Endothelial immunothrombotic modulation	Recombinant human soluble thrombomodulin	Thrombin binding; protein C activation; attenuation of endothelial inflammatory activation	Reduced thrombin amplification, microvascular coagulation, and endothelial injury	Clinical use in Japan; SCARLET trial; meta-analytical evidence with heterogeneous mortality effects	DIC resolution; organ dysfunction; bleeding; mortality
Anticoagulant/endothelial protection	UFH/LMWH	Antithrombin-mediated anticoagulation; modulation of immunothrombosis; mitochondrial and endothelial protection	Reduced microthrombosis, endothelial injury, and perfusion failure	Observational clinical data; meta-analyses; experimental mechanistic evidence	Mortality; bleeding; SIC/DIC scores; platelet count; coagulation parameters
Endothelial stabilization	Defibrotide	Anti-inflammatory, antithrombotic, profibrinolytic, antiapoptotic, and angioprotective effects	Reduced endothelial activation, platelet adhesion, vWF release, and vascular leakage	Experimental and translational evidence	Endothelial activation markers; vascular leakage; organ dysfunction
Precision immunomodulation	Tocilizumab/IL-6 blockade	IL-6 receptor inhibition; attenuation of cytokine-driven endothelial activation	Reduced endothelial hyperactivation, capillary leakage, and immunothrombotic amplification in selected hyperinflammatory phenotypes	Limited clinical observations; translational rationale	Inflammatory markers; hemodynamic stabilization; organ dysfunction; safety

↑ indicates an increase, enhancement, or upregulation; ↓ indicates a decrease, reduction, or inhibition of the corresponding biological process, pathway, or clinical parameter. **Abbreviations:** AKI, acute kidney injury; ANP, atrial natriuretic peptide; AST, aspartate aminotransferase; ALT, alanine aminotransferase; BUN, blood urea nitrogen; CLP, cecal ligation and puncture; DIC, disseminated intravascular coagulation; EPC, endothelial progenitor cell; ERK, extracellular signal–regulated kinase; ETA/ETB, endothelin receptor A/B; FFP, fresh frozen plasma; GAG, glycosaminoglycan; HMGB1, high-mobility group box 1; ICU, intensive care unit; IL-6, interleukin 6; MAP, mean arterial pressure; MK2, MAPK-activated protein kinase 2; NE, norepinephrine; Rac, Ras-related C3 botulinum toxin substrate; RAGE, receptor for advanced glycation end products; ROS, reactive oxygen species; S1P, sphingosine-1-phosphate; S1P1, sphingosine-1-phosphate receptor 1; SOFA, Sequential Organ Failure Assessment; VCAM-1, vascular cell adhesion molecule 1; UFH, unfractionated heparin; LMWH, low-molecular-weight heparin; SIC, sepsis-induced coagulopathy; vWF, von Willebrand factor.

## Data Availability

Not applicable.

## Abbreviations

ARDS	Acute Respiratory Distress Syndrome
APC	Article Processing Charge
AST	Aspartate Aminotransferase
ALT	Alanine Aminotransferase
BUN	Blood Urea Nitrogen
CLP	Cecal Ligation and Puncture
CFU-EPCs	Colony-Forming Units of Endothelial Progenitor Cells
C/EBPβ	CCAAT/Enhancer Binding Protein Beta
DAMPs	Damage-Associated Molecular Patterns
DIC	Disseminated Intravascular Coagulation
eNOS	Endothelial Nitric Oxide Synthase
EPCs	Endothelial Progenitor Cells
HMGB1	High-Mobility Group Box-1
ICAM-1	Intercellular Adhesion Molecule-1
IFN-γ	Interferon Gamma
IL-1β	Interleukin 1 Beta
IL-6	Interleukin 6
IL-10	Interleukin 10
LPS	Lipopolysaccharide
MAPK	Mitogen-Activated Protein Kinase
MLCK	Myosin Light Chain Kinase
MMP14	Matrix Metalloproteinase 14
MRSA	Methicillin-Resistant *Staphylococcus aureus*
NETs	Neutrophil Extracellular Traps
NF-κB	Nuclear Factor Kappa B
NO	Nitric Oxide
NT-proBNP	N-terminal prohormone of Brain Natriuretic Peptide
PAMPs	Pathogen-Associated Molecular Patterns
PAI-1	Plasminogen Activator Inhibitor-1
PBS	Phosphate-Buffered Saline
Prg4	Proteoglycan 4 (Lubricin)
RAGE	Receptor for Advanced Glycation End Products
RhoA	Ras Homolog Family Member A
ROS	Reactive Oxygen Species
SDF-1	Stromal Cell-Derived Factor 1
S1P	Sphingosine-1-Phosphate
SOFA	Sequential Organ Failure Assessment
TEER	Transendothelial Electrical Resistance
TFPI	Tissue Factor Pathway Inhibitor
TLR/TLR4	Toll-like Receptor/Toll-like Receptor 4
TNF-α	Tumor Necrosis Factor Alpha
TRPM7/TRPV4	Transient Receptor Potential Melastatin/Vanilloid Channels
VCAM-1	Vascular Cell Adhesion Molecule-1
VE-cadherin	Vascular Endothelial Cadherin
VEGF	Vascular Endothelial Growth Factor
VEGFR	Vascular Endothelial Growth Factor Receptor
ZO-1	Zonula Occludens-1
